# Implications for nitrogen and sulphur cycles: phylogeny and niche-range of *Nitrospirota* in terrestrial aquifers

**DOI:** 10.1093/ismeco/ycae047

**Published:** 2024-03-27

**Authors:** Olivia E Mosley, Emilie Gios, Kim M Handley

**Affiliations:** School of Biological Sciences, The University of Auckland, Auckland 1010, New Zealand; Present address: NatureMetrics Ltd, Surrey Research Park, Guildford GU2 7HJ, United Kingdom; School of Biological Sciences, The University of Auckland, Auckland 1010, New Zealand; Present address: NINA, Norwegian Institute for Nature Research, Trondheim 7034, Norway; School of Biological Sciences, The University of Auckland, Auckland 1010, New Zealand

**Keywords:** Nitrospirota, aquifer, groundwater, nitrogen-cycling, sulphur-cycling

## Abstract

Increasing evidence suggests *Nitrospirota* are important contributors to aquatic and subsurface nitrogen and sulphur cycles. We determined the phylogenetic and ecological niche associations of *Nitrospirota* colonizing terrestrial aquifers. *Nitrospirota* compositions were determined across 59 groundwater wells. Distributions were strongly influenced by oxygen availability in groundwater, marked by a trade-off between aerobic (*Nitrospira*, *Leptospirillum*) and anaerobic (*Thermodesulfovibrionia*, unclassified) lineages. Seven *Nitrospirota* metagenome-assembled genomes (MAGs), or populations, were recovered from a subset of wells, including three from the recently designated class 9FT-COMBO-42-15. Most were relatively more abundant and transcriptionally active in dysoxic groundwater. These MAGs were analysed with 743 other *Nitrospirota* genomes. Results illustrate the predominance of certain lineages in aquifers (e.g. non-nitrifying *Nitrospiria*, classes 9FT-COMBO-42-15 and UBA9217, and *Thermodesulfovibrionales* family UBA1546). These lineages are characterized by mechanisms for nitrate reduction and sulphur cycling, and, excluding *Nitrospiria*, the Wood–Ljungdahl pathway, consistent with carbon-limited, low-oxygen, and sulphur-rich aquifer conditions. Class 9FT-COMBO-42-15 is a sister clade of *Nitrospiria* and comprises two families spanning a transition in carbon fixation approaches: f_HDB-SIOIB13 encodes rTCA (like *Nitrospiria*) and f_9FT-COMBO-42-15 encodes Wood–Ljungdahl CO dehydrogenase (like *Thermodesulfovibrionia* and UBA9217). The 9FT-COMBO-42-15 family is further differentiated by its capacity for sulphur oxidation (via DsrABEFH and SoxXAYZB) and dissimilatory nitrate reduction to ammonium, and gene transcription indicated active coupling of nitrogen and sulphur cycles by f_9FT-COMBO-42-15 in dysoxic groundwater. Overall, results indicate that *Nitrospirota* are widely distributed in groundwater and that oxygen availability drives the spatial differentiation of lineages with ecologically distinct roles related to nitrogen and sulphur metabolism.

## Introduction

The microbial N and S cycles are catalyzed by diverse microorganisms in terrestrial aquifers [1–3]. Their intersection occurs in bacteria that couple sulphur oxidation with nitrogen reduction [4–6], which may be favourable in characteristically organic carbon-poor groundwater [6, 7]. Nitrogen species are usually present in low concentrations in groundwater, particularly nitrite and ammonia, which are unstable and easily convert to nitrate through microbial nitrification [8]. Excess nitrogen concentrations stem primarily from anthropogenic sources [9]. Denitrification is the primary process for nitrate attenuation in groundwater [10]. The rate of N loss is usually dependent on the availability of dissolved organic carbon for heterotrophic denitrification [11]. However, given limited dissolved organic carbon [6], autotrophic denitrification is likely an ecologically important process in groundwater.

Autotrophic denitrifiers use inorganic electron donors, such as reduced sulphur, Fe(II), and H_2_ in groundwater [5]. Sulphur can be plentiful in aquifers and is typically present as sulphate, bisulphide, or hydrogen sulphide [12], with sulphate concentrations ranging from 0 to 230 mg/L [13]. Reduced forms (e.g. hydrogen sulphide, iron sulphide precipitate) or partially reduced forms (elemental sulphur, sulphite, and thiosulphate) can derive from the activity of sulphate-reducing ± sulphur-oxidizing bacteria [12, 14]. Sulphur oxidation is among the most energetically favourable processes for chemoautotrophs (transferring up to eight electrons per sulphur atom) [4] and releases almost twice as much kJ per mol electron donor than iron oxidation [15]. Recent research suggests the functional capacity for autotrophic sulphur (and nitrogen) oxidation in aquifers can be substantial [7]. Nonetheless, sulphur oxidation remains understudied across the large differences in oxygen, nitrate, and dissolved organic carbon (DOC) concentrations found in groundwater [3].

*Nitrospirota* (or *Nitrospirae*) are widely reported to contribute to aerobic nitrification in aquatic sediments [16, 17], including aquifers [18, 19]. However, anaerobic sulphur and nitrogen metabolism may be important features of this phylum in subsurface environments [20–22]. Although the phylum is typified by chemolithoautotrophic aerobes capable of nitrification (i.e. *Nitrospira*) [23, 24], Mn(II) oxidation (*Candidatus* Manganitrophaceae) [25], and iron oxidation (*Leptospirillum*) [26], members of the less-studied *Thermodesulfovibrionia* class (*Thermodesulfovibrio* spp.) reduce nitrate and/or sulphate [27] or are predicted to oxidize sulphur (*Candidatus* Magnetobacterium) [28]. Recent research also suggests novel *Nitrospirota* from terrestrial aquifers could oxidize sulphur [21, 22], reduce nitrate, and fix carbon [21]. Phylogenomic analyses further indicate that anaerobic nitrogen and sulphur metabolism is an ancestral feature retained by *Nitrospirota* in the marine and terrestrial subsurface [20]. Given the prevailing characteristics of the phylum, *Nitrospirota* may represent a reservoir of novel nitrogen- and sulphur-cycling autotrophs in groundwater ecosystems.

Here, we determined the distribution of *Nitrospirota* lineages associated with oxic to anoxic terrestrial groundwater ecosystems. Groundwater samples, encompassing large differences in nitrate, oxygen, and DOC concentrations, were collected for 16S rRNA gene amplicon analysis to determine the diversity and distribution of *Nitrospirota*. Metagenome-assembled genomes (MAGs), reconstructed from eight groundwater wells, included seven *Nitrospirota* MAGs paired with transcriptional data. These seven and 743 other *Nitrospirota* and Nitrospirota_A genomes were compared to determine the relationship between groundwater lineages and carbon, nitrogen, and sulphur metabolisms. Analyses show that certain lineages of *Nitrospirota* are common to aquifers, occupying distinct niches governed by oxygen availability, and illustrate metabolic divergences among groundwater-derived lineages.

## Materials and methods

### Samples for amplicon and omic analyses

Amplicon and omic data, as well as accompanying geochemical profiles, were generated from groundwater samples as described previously [3, 29]. In brief, 80 filtered groundwater samples (biomass ≥0.22 μm) were collected from 59 wells across 10 aquifers (Auckland, Waikato*,* Wellington, and Canterbury regions, New Zealand). All were used for amplicon analyses. A subset of 16 samples from eight wells across two alluvial aquifers in Canterbury were used for metagenomics (samples gwj01–gwj16) and six for metatranscriptomics (samples gwj09, gwj11, and gwj13–gwj16 with RNA integrity numbers ≥6% or 30% of fragments >200 nucleotides [3]). The two samples per well were collected sequentially: first, filtered groundwater, and, second, biofilm-enriched filtered groundwater following low-frequency sonication [29].

Amplicon sequence variants (ASVs) were generated using the QIIME2 dada2 denoise-paired command in QIIME2 (2022.2), with 10 bp removed from the start of reads and truncation at 230 bp [30]. Classification used the feature-classifier classify-sklearn command and the QIIME2 pretrained classifier silva-138-99-515-806-nb-classifier.qza derived from the SILVA-138 database [31]. In R v4.2.1, non-prokaryotic sequences were removed, leaving 44 276 from an initial 46 713 ASVs, and rarefied to the minimum sample depth (3104) with phyloseq rarefy_even_depth (bioconductor 3.16) [32].

Trimmed metagenomic data from the 16 Canterbury samples were assembled to generate 396 unique MAGs (70%–100% completion, 0%–5% contamination, dereplication threshold, 99% average nucleotide identity, ANI) using methods described previously [29]. Quality was assessed using checkM v1.012 [33], and dereplication was performed using dRep v2.0.5 [34]. Trimmed metagenomic and metatranscriptomic reads were mapped to MAGs to determine genome coverage and gene expression outlined previously [29] (summary in Supplementary Information). Genome sizes were estimated by (bin size − (bin size * contamination))/(completeness) [35].

### Average nucleotide identities and phylogenetic core gene trees

Comparative analyses used all 750 available *Nitrospirota* genomes with < 5% estimated contamination in the Genome Taxonomy Database (GTDB, release 214), including Nitrospirota_A and Nitrospirota_B, collectively referred to as *Nitrospirota* below, and seven *Nitrospirota* MAGs we reconstructed (Table S1). The total available pool of *Nitrospirota* genomes available in the 214 release before filtering was 802 (also see Table S1). Pairwise ANIs were determined using fastANI v1.33 [36]. Core gene alignments were undertaken using GTDB-Tk v2.1.1. Concatenated alignments (based on 120 bacteria marker genes and 5036 amino acids, Table S2) were used to construct a maximum-likelihood phylogenomic tree in IQ-TREE v2.2.2.2 [37] using the ModelFinder [38] best-fit non-taxa-specific model LG + F + I+ with 1000 ultrafast bootstrap replicates (see Fig. S1 for tree with bootstrap values). Tree construction was repeated as above using 1000 standard bootstrap replicates, and a core gene alignment based on a subset of 115 *Nitrospirota* genomes representing all seven classes and 19 orders (with 75 representatives from the 30 families with genomes from terrestrial aquifers) (Fig. S1). Consensus trees were rooted to representatives of 11 different phyla (Table S1).

### Metabolic predictions

First, metabolic predictions based on protein-coding gene sequences were carried out on eight *Nitrospirota* MAGs we reconstructed from groundwater samples, as summarized here and described previously [29]. Protein-coding gene sequences for MAGs were predicted using Prodigal v2.6.3 [39]. Predicted protein-coding sequences were annotated using USEARCH v9.02132 [40] with the usearch_global command (−id 0.5 –e-value 0.001 –maxhits 10), UniRef100 [41], UniProt [42], and KEGG databases [43]. Additional hidden Markov model (HMM) searches were also carried out on MAGs and reference genomes using HMMER v3.3 [44] against Pfam [45], TIGRfam [46] databases, and databases with HMM cutoffs provided by Anantharaman *et al.* [1]. Additionally, protein sequences were searched against the eggNOG v5.0 database [47] using eggNOG-mapper v2 [48] with default parameters. Second, for comparative analyses with other publicly available *Nitrospirota* genomes, metabolic predictions of all 750 *Nitrospirota* genomes used for the phylogenomic tree (including MAGs derived from this study) were undertaken with DRAM v1.3.5 with default settings [49]. The functional assignments of key metabolic genes identified by these two sets of annotations were interrogated further and validated, as described below.

Protein sequences were searched against representative sequences from the NCBI Protein database using BLASTp [50] to: (i) compare dissimilatory sulphite reductase (DsrAB) sequences with oxidative and reductive types and classify genes into either type, (ii) identify additional nitrite oxidoreductase (*nxrAB*) and nitrate reductase (*narGH*) genes, and (iii) confirm missing citric acid (TCA) cycle genes (filtering criteria: sequence identity >30%, e-value < 0.001, pairwise alignment length > 70%; Table S3). To further validate functional assignments, protein sequences - for NosZ, Dsr(A)B, NxrA, and NarG - trees were aligned using MUSCLE v5 [51] with default parameters. Reference sequences for alignments were obtained from UniProt [42] and NCBI [52] protein databases. Alignments were trimmed using trimAl with default parameters [53] for DsrAB, NxrA, and NarG, or using Geneious v11.1.2 for NosZ (https://www.geneious.com), guided by the secondary structure of NosZ from *Paracoccus denitrificans* (Protein Database entry 1FWX) [54]. Maximum-likelihood trees were constructed as for the phylogenomic tree. All trees were annotated with iTOL [55]. Signal peptides in nitrous-oxide reductase (NosZ) sequences were detected using SignalP-5.0 [56]. Protein domain searches using NosZ, NxrA, and NarG sequences in class 9FT-COMBO-42-15 genomes were undertaken using the Conserved Domain Database (e-value 0.001) [57]. IslandViewer 4 [58] was used to identify genomic islands in MAG nzgw271.

### Statistical analyses

All statistical analyses were carried out using R version 4.0.3. Bray–Curtis dissimilarities for distance-based redundancy analysis (db-RDA) used ASV relative abundances with vegan v2.5.6 [59].

## Results and discussion

### Phylogenetically diverse *Nitrospirota* found in aquifers

**Figure 1:**
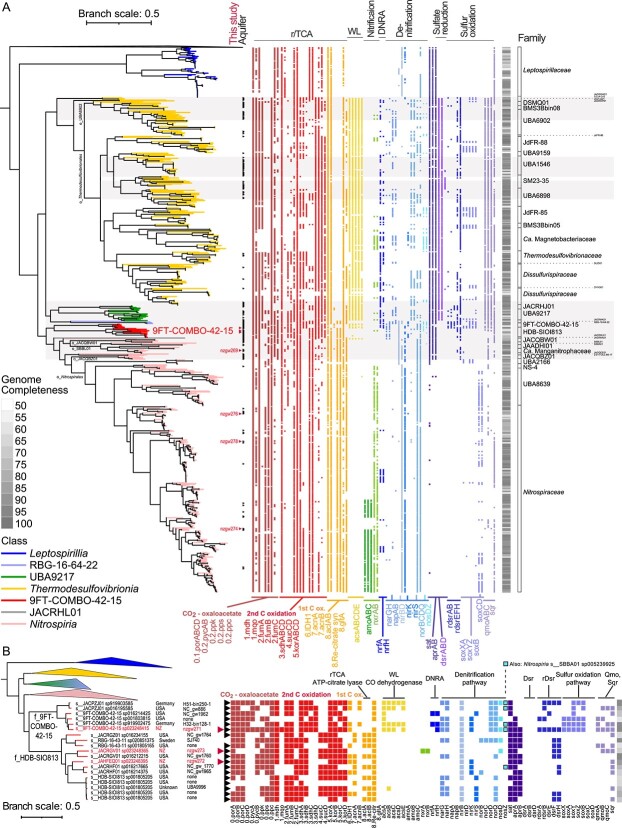
Maximum likelihood phylogenomic tree of 750 *Nitrospirota* and their genomically inferred capacity for carbon metabolism, nitrogen and sulphur cycling; (A) full tree with branches coloured by class. Grey shading denotes parts of the tree largely comprising genomes derived from terrestrial aquifers; (B) tree highlighting the two orders of class 9FT-COMBO-42-15 (upper clade = order 9FT-COMBO-42-15; lower clade = order HDB-SIOI1813) with other classes collapsed; branch labels give GTDB species designations, source country and NCBI strain identifiers; in (A) and (B), red font and arrows indicate MAGs from this study. Genes present are indicated by coloured squares to the right: genes associated with the TCA/rTCA cycles (dark red = CO_2_ to oxaloacetate; red = second C oxidation pathway; orange = first), Wood–Ljungdahl pathway CO dehydrogenase (yellow), comammox/nitrification (green), DNRA (dark blue), denitrification (different blue shades denote genes associated nitrate reductases, nitrite reductases, nitric oxide reductase, and nitrous oxide reductase), sulphate reduction or oxidation (different purple shades denotes genes associated with ATP-sulfurylase and adenylyl-sulphate reductases, dissimilatory sulphite reductase, reverse dissimilatory reductase, the sulphur oxidation pathway, quinone-modifying oxidoreductase, and sulphide-quinone oxidoreductase); only TCA/rTCA genes annotated in at least one genome are shown, and numbers prefixing gene names indicate steps (0–8) from CO_2_ to oxaloacetate to malate, etc., scale bars represent number of substitutions per site; coloured squares with black outlines in (B) denote lactonase domain encoding *nosZ* like genes; bootstrap support is shown in Fig. S1; acronym: *Re-citrate syn*, Re-citrate synthase.

To determine the phylogenetic distribution of *Nitrospirota* lineages in aquifers, we undertook an analysis of the phylum using 750 genomes. These spanned 7 classes, 19 orders, and 42 families (Table S1, ≤ 5% contamination in GTDB), and included seven *Nitrospirota* MAGs (nzgw269, 271–274, 276, 278) from a pool of 626 unique MAGs we generated from eight oxic or dysoxic groundwater wells [3]. Three of the seven were classified as *Nitrospirota* class 9FT-COMBO-42-15 (two HDB-SIOI813 families; one 9FT-COMBO-42-15 family). Four were *Nitrospiria* (one *Manganitrophaceae*; three *Nitrospiraceae*). None were Nitrospirota_A (*Leptospirillia*). A *Nitrospira* complete ammonia-oxidizer (comammox) MAG was obtained (nzgw279 [3]), but excluded from the phylogenetic analysis (completeness 73.02%, contamination 5.45%). A phylogenetic tree based on GTDB core gene alignments showed three major phylogenetic clusters comprising classes: (i) *Leptospirillia*; (ii) *Thermodesulfovibrionia*, UBA9217, and RBG-16-64-22; and (iii) 9FT-COMBO-42-15 and *Nitrospiria* (Fig. 1A and Fig. S1). Lineages in the second cluster are predicted to be basal or ancestral to *Nitrospiria* [20], whereas 9FT-COMBO-42-15 has an intermediate phylogenetic placement between the basal lineages and *Nitrospiria* (Fig. 1A).

**Figure 2:**
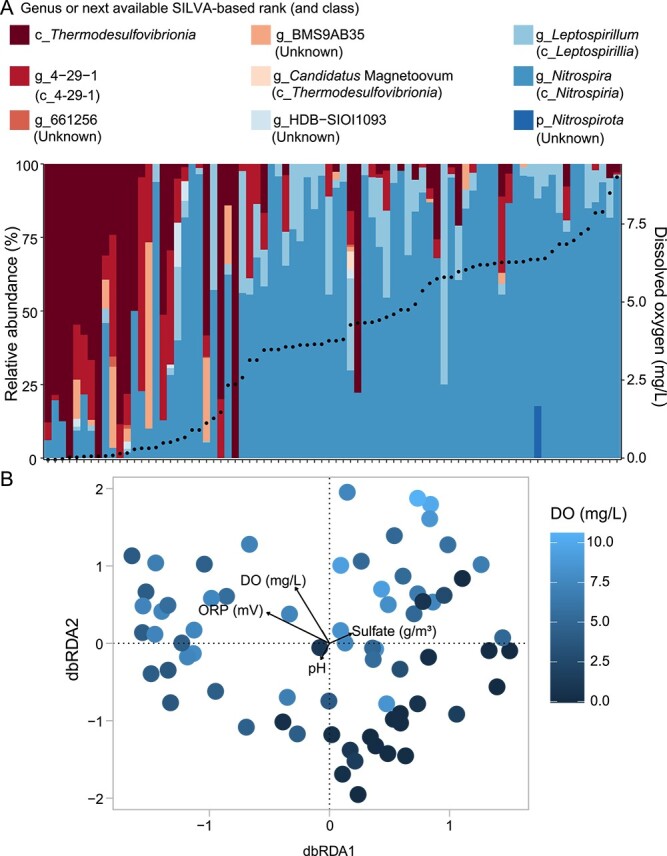
Stacked bar graph and ordination plot showing the environmental variables and factors influencing the structure of the *Nitrospirota* (and Nitrospirota_A) groundwater community based on 16S rRNA gene amplicon data. (A) Composition of the *Nitrospirota* community across 80 samples, ordered by dissolved oxygen (DO) concentrations. Black points within plot = DO content. Bars represent different genera. Black font = SILVA classifications (genus level or next rank with a classification). Based on GTDB small subunit rRNA gene BLAST bast comparisons, SILVA classes shown correspond to the same GTDB classes, except for: c_661256, c_BMS9AB35, and c_HDB-SIOI1093 (GTDB classes unknown), and c_4-29-1 (GTDB classes 9FT-COMBO-42-15, UBA9217, and *Thermodesulfovibrionia*) (Table S1). Acronyms: *G*, genus; *c*, class; *p*, phylum. (B) Distance-based redundancy analysis of the 16S amplicon data based on a Bray–Curtis dissimilarity matrix of ASV relative abundances. Each point in the ordination represents the *Nitrospirota* and Nitrospirota_A community composition in each sample, and is shaded dark to light blue based on increasing groundwater DO content. Vectors show environmental variables that significantly (*P* < 0.05) constrain the variability in community composition.

*Nitrospirota* genomes derived from terrestrial aquifers were found across most taxonomic classes (six out of seven in GTDB). Representatives of *Leptospirillia* were lacking, although 16S rRNA gene data indicate the presence of this lineage in oxic groundwater (Fig. 2A). Instead, groundwater genomes included those from canonical nitrifiers and comammox bacteria [3], and most were derived from poorly studied and populated classes. Of these, certain taxonomic groups were almost entirely represented by MAGs recovered from aquifers: deep-branching *Nitrospiria* (earlier branching orders than *Nitrospirales*), class 9FT-COMBO-42-15, class UBA9217, four genomes comprising class RBG-16-64-22, and clusters of *Thermodesulfovibrionia* spanning multiple families in both the major orders *Thermodesulfovibrionales* (notably families UBA1546, SM23-35, and UBA6898) and UBA6902 (grey-shading in Fig. 1A, Table S1). The broad geographic distribution of aquifers that these over-represented taxonomic groups were reproducibly found across (e.g. different U.S. states, Germany, New Zealand) suggests these taxa are particularly well-adapted to aquifer conditions. For example, class 9FT-COMBO-42-15 almost exclusively comprises uncultivated taxa recently sampled from aquifers in the USA (Colorado [1] and California [60]), Germany (Thuringia [21]), and New Zealand (our sampling, Canterbury [3]) (18 out of 19 genomes, Fig. 1B, Table S1).

The two families that currently comprise class 9FT-COMBO-42-15 (9FT-COMBO-42-15 and HDB-SIOI813) encompass 8 GTDB designated genera and 15 different species (Table S1). The 9FT-COMBO-42-15 family comprises genus 9FT-COMBO-42-15 (four species sharing 85%–88% ANI) and JACPZJ01 (two sharing 94% ANI), with ANIs for genus 9FT-COMBO-42-15 well below the proposed species cutoff of 95% [61] (Table S4). All are known from MAGs from groundwater or aquifer sediment (MAG nzgw271, this study and [3], Germany x2 [21], USA x3 [1, 60]). Similarly, 12 of the 13 members of family HDB-SIOI813 derive from aquifers (USA, New Zealand, and unknown), with MAGs nzgw272–nzgw273 deriving from this study. Aquifer-derived HDB-SIOI813 taxa encompass six genera and eight species (78%–92% intra-genus ANI among species or 97%–99% ANI among the same species). Of the 18 members of the class from aquifers, four are represented by genomes with >90% estimated completeness: family 9FT-COMBO-42-15 genus 9FT-COMBO-42-15 (MAG nzgw271, 95% completeness, and MAG Hainich-H32-bin128–1, 94%) and JACPZJ01 (MAG Hainich-H51-bin250-1, 94%), and family HDB-SIOI813 genus JACRGV01 (MAG NC_groundwater_1760_Pr3_B-0.1um_42_52, 95%) (Fig. 1B, Table S1). Members of this, and the other aquifer-adapted lineages, represent a substantial underexplored fraction of the *Nitrospirota*, whose metabolic attributes are currently accessible only through environmental omic data.

### Spatial differentiation of *Nitrospirota* lineages in groundwater based on dissolved oxygen content

To explore the spatial distribution of *Nitrospirota* in aquifers, and its association with water chemistry, we analysed 16S rRNA gene amplicon sequences classified as *Nitrospirota* from 59 geographically widespread wells. Results showed proportionally more unclassified *Thermodesulfovibrionia* and genus 4-29-1 (SILVA taxonomy) at low oxygen sites, and more *Nitrospira*, and, to a lesser extent, *Leptospirillum*, at high oxygen sites (Fig. 2A). The relationship between 4-29-1 and GTDB-designated taxonomies is unclear, but 16S rRNA gene based sequence similarities are shared between the 4-29-1 class and GTDB classes 9FT-COMBO-42-15, UBA9217, and *Thermodesulfovibrionia* (Table S1). The presence of 4-29-1 could potentially indicate the presence of any of these lineages (e.g. 9FT-COMBO-42-15, which was sampled via metagenomics from some of the same samples; see discussion below). The spatial distribution of *Nitrospirota* taxa was significantly associated with changes in dissolved oxygen and ORP (Fig. 2B), whether aquifers were confined or unconfined, aquifer location, sample type (groundwater, biomass-enriched groundwater), sulphate, and pH, which collectively explained 9% (*R*^2^ adjusted) of variation in *Nitrospirota* community composition (db-RDA permutation-test, *P <* 0.05, permutations = 999, Table S5). *Nitrospirota* are therefore present in diverse aquifers, with distinct environmental niches, such as those governed by oxygen availability, occupied by different lineages.

**Figure 3:**
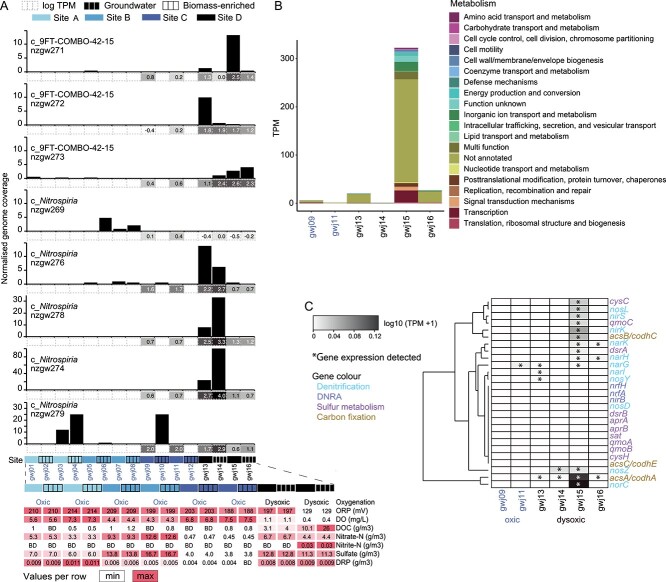
Relative abundance and gene expression of *Nitrospiria* MAG nzgw271 across different geochemical conditions at sites A–D. (A) Bar plots showing the relative abundance of *Nitrospirota* MAGs based on genome coverages normalized to library size across each site (samples right to left: gwj01–gwj16) and sample type (groundwater and biomass-enriched groundwater). An eighth MAG (comammox *Nitrospira* nzgw279, 5.45% contamination, Table S1) is included [3]. Groundwater and biomass-enriched groundwater (i.e. groundwater collected post sonication to detach aquifer biofilms and particles) were obtained sequentially from the same groundwater wells. Geochemical parameters per site/sample are given below the bar plot (abbreviations: ORP, oxidation–reduction potential; DO, dissolved oxygen; DOC, dissolved organic carbon; DRP, dissolved reactive phosphorus). (B) Functional categories (eggNOG) of all transcribed genes predicted from MAG nzgw271. (C) Abundance of nitrogen and sulphur cycling transcripts per million (TPM) across each sample. Asterisks indicate presence of transcripts (including where expression was relatively low).

Broadly similar results were obtained based on the eight MAGs recovered from a subset of sampled aquifers. Comammox *Nitrospira* nzgw279 was proportionally most abundant in oxic groundwater, whereas members of the 9FT-COMBO-42-15 class (MAGs nzgw271–273), and most earlier-branching and poorly characterized *Nitrospiria* (MAGs nzgw274, nzgw276, and nzgw278) were more abundant under dysoxic conditions (excluding *Candidatus* Manganitrophus nzgw269, Fig. 3A). As indicated above, class 9FT-COMBO-42-15 represents one of the *Nitrospirota* lineages over-represented in terrestrial aquifers (Fig. 1A). Representatives of both families in the class (9FT-COMBO-42-15 and HDB-SIOI813) were relatively much more abundant and exhibited higher relative gene expression in dysoxic versus oxic groundwater (MAG nzgw271, genus 9FT-COMBO-42-15; HDB-SIOI813 MAGs nzgw272 and nzgw273, genus JAHFEQ01 and JACRGV01; Fig. 3A–C). Both families also share the capacity for nitrate reduction (via NarGH) and nitrite reduction to NO (via NirK or NirS) (Fig. 1A) [62], and hence anaerobic metabolism. Similarly, another member of the 9FT-COMBO-42-15 family (MAG H51-bin250-1, genus JACPZJ01) was found to dominate suboxic-anoxic groundwater elsewhere (Hainich Critical Zone Exploratory, Germany) [21]. Results therefore indicate members of class 9FT-COMBO-42-15 are adapted to low-oxygen groundwater habitats.

### Metabolic trait differences among *Nitrospirota* in terrestrial aquifers

Collectively, *Nitrospirota* lineages, including the diversity of lineages found in aquifers, are associated with aerobic, microaerobic, and anaerobic lifestyles, and the transformation of nitrogen, sulphur, iron, and manganese species for energy gain. Well-characterized genera, *Nitrospira* (class *Nitrospiria*) and *Leptospirillum* (class *Leptospirillia*), are capable of autotrophic and aerobic nitrite oxidation, comammox, or microaerobic-anaerobic iron oxidation [24, 26], and dominated *Nitrospirota* in oxic groundwaters in this study (Fig. 2A). *Candidatus* Manganitrophus (*Nitrospiria*), which couples manganese oxidation with aerobic respiration [25], was also observed in groundwater (MAG nzgw269, oxic groundwater in this study, and two locations in the USA, Table S1). However, most genomes derived from aquifers to date are characterized by genes associated with nitrate reduction and sulphur metabolism (Fig. 1A) and are suggestive of anaerobic (or microaerobic) lifestyles (e.g. members of the 9FT-COMBO-42-15 class). In addition, magnetotactic *Thermodesulfovibrionia*, such as *Candidatus* Magnetoovum (from a partially oxic sample, Fig. 2A), are thought to oxidize sulphur as aerotolerant anaerobes or microaerobes [63]. Moreover, *Thermodesulfovibrio* (reported from a couple of groundwater sites) are heterotrophic sulphate reducers [64, 65], and hence anaerobic, or are suggested to couple sulphate reduction to C1 metabolism [20].

To further illustrate the metabolic traits characteristic of *Nitrospirota* lineages alongside those over-represented in terrestrial aquifers, we searched for genes associated with carbon fixation and nitrogen and sulphur cycling in the 750 genomes. Results highlight the distinct metabolic strategies encoded by later-branching *Nitrospiria* (i.e. aerobic nitrification and rTCA-based carbon fixation [23, 66]) versus most other *Nitrospirota* lineages (i.e. anaerobic/microaerobic sulphur and nitrogen metabolism, and Wood–Ljungdahl (WL) CO dehydrogenase) (Fig. 1A), consistent with a recent phylogenomic analysis of the phylum [20]. Genomes derived from groundwater to date are largely associated with WL-dependent lineages (i.e. classes UBA9217, 9FT-COMBO-42-15, and *Thermodesulfovibrionia*). Although those associated with the intermediate class, 9FT-COMBO-42-15, span both WL-dependent and WL-independent groups. In addition, the four available RBG-16-64-22 genomes lack the WL pathway, and some very early branching non-nitrifying *Nitrospiria* orders that lack the WL pathway are also largely comprised of aquifer-derived genomes (grey-shading, Fig. 1A). Overwhelmingly, lineages over-represented in terrestrial aquifers (including early branching *Nitrospiria*) are characterized by genes encoding for sulphate reduction (dissimilatory sulphite reductase, DsrAB), sulphur oxidation (rDsrAB or the sulphur oxidation pathway, Sox), dissimilatory nitrate reduction to ammonium (DNRA), and/or the denitrification pathway. The metabolic properties of these over-represented lineages [20] highlight the tendency for aquifers to be oxygen-depleted and sulphur-rich (Fig. 3A) [3], and consequently rich in sulphur-cycling taxa [1].

### Metabolic transitions within class 9FT-COMBO-42-15

Results show a transition in carbon fixation mechanisms within the class 9FT-COMBO-42-15, from WL CO dehydrogenase in the 9FT-COMBO-42-15 family to rTCA in family HDB-SIOIB13 (the only families of their respective orders, Fig. 1B). The same transition is repeated within the clade encompassing classes UBA9217 and RBG-16-64-22, although the latter is poorly represented by available genomes. These transitions reflect the rTCA dependence in later-evolved *Nitrospiria*, and the phylogenetic relatedness of class 9FT-COMBO-42-15 to *Nitrospiria* (Fig. 1A). *Nitrospiria*, HDB-SIOIB13, and RBG-16-64-22 are characterized by the presence of *aclAB* (ATP citrate lyase) genes lacking from the 9FT-COMBO-42-15 family and the UBA9217 and *Thermodesulfovibrionia* classes, which encode incomplete rTCA cycles (Fig. 1A). These other lineages (excluding *Leptospirillia*) instead encode WL *acsABCDE* CO dehydrogenase genes. The 9FT-COMBO-42-15 and HDB-SIOI813 families are also distinguished by their capacities for anaerobic nitrogen metabolism and sulphur metabolism. Notably, 9FT-COMBO-42-15 harbours an expanded repertoire of genes for sulphur oxidation, as discussed further below (Fig. 1B).

Members of the 9FT-COMBO-42-15 family potentially fix carbon via the WL pathway to support autotrophic sulphur oxidation, as suggested for cable bacteria [67]. WL-based carbon fixation was recently suggested for groundwater-derived MAG, H51-bin250-1 (JACPZJ01 genus, 9FT-COMBO-42-15 family) [21], and for *Candidatus* Magnetobacterium [68]. Analysis of all members of the 9FT-COMBO-42-15 family shows WL bacterial *acsABCDE* genes are universally present (Fig. 1B). AcsABCDE incorporates carbon dioxide into the carboxylic group of acetate, combining CO from the western branch with methyl from the eastern branch [69]. Inspection of MAG nzgw271 (genus 9FT-COMBO-42-15, recovered from dysoxic groundwater sample gwj15, Fig. 3A) shows key western and eastern branch genes were present (Fig. 1B, see Supplementary Information). Transcript analysis further shows that CO dehydrogenase (*codhA*/*acsA*) and acetyl-CoA synthase (*codhC*/*acsB*) were actively expressed by nzgw271, alongside genes for the denitrification pathway and sulphur oxidation (Fig. 3C), suggesting autotrophic nitrate-dependent sulphur oxidation. In contrast, *Thermodesulfovibrionia* are suggested to use the WL pathway for C1 metabolism (formate and CO oxidation to CO_2_) [20], consistent with the reverse operation of the WL pathway by sulphate reducers [70], and the predicted prevalence of sulphate reducers among the *Thermodesulfovibrionia* (Fig. 1A) [20]. This implies distinct roles for the WL pathway between *Thermodesulfovibrionia*, which dominated suboxic-anoxic groundwater in this study (Fig. 2A), and 9FT-COMBO-42-15 (e.g. nzgw271), which were likewise most abundant in low-oxygen groundwater communities (Fig. 3A).

Although rTCA carbon fixation is employed by sulphide-oxidizers in anoxic groundwater [14], the incomplete rTCA cycle encoded by members of the 9FT-COMBO-42-15 family likely functions to biosynthesize intermediates and replenish the oxaloacetate pool that condenses with acetyl-CoA (Fig. 1, Supplementary Information). Incomplete TCA and rTCA cycles can still convert pyruvate to necessary biosynthetic intermediates under anaerobic or microaerophilic conditions. They are suggested to be from an intermediate stage in the evolution of the oxidative catabolic TCA cycle that was primarily for amino acid biosynthesis, and comprise a non-cyclic “horseshoe” pathway lacking the oxidative enzymatic step to form succinate (catalyzed by succinyl-CoA synthetase, SucCD, in the rTCA cycle) (Fig. 1B, Supplementary Information) [71].

### Versatile nitrogen and sulphur metabolism of class 9FT-COMBO-42-15

#### Capacity for both ammonification and denitrification

Collectively, members of the 9FT-COMBO-42-15 class, have several nitrogen-cycling genes, such as those encoding nitrate (NarGH) and nitrite reductases (NrfAH, NirB, NirK, and NirS), nitric oxide reductase (NorBC), and nitrous oxide reductase (NosZ or NosZ-like) (Fig. 1B, Table S6). Their presence suggests the capacity to utilize at least part of the denitrification pathway, from nitrate to N_2_, is a common feature of the class, and that members of the 9FT-COMBO-42-15 family (genus 9FT-COMBO-42-15) can additionally catalyze DNRA.

Most members of the class harbour genes encoding NarGH respiratory nitrate reductases (42%–44% amino acid identity with NarG from *Escherichia coli*, Table S7). For example, MAG nzgw271 harbours a respiratory nitrate reductase *narGHI* gene cluster, and a co-localized *narK* gene for nitrate/nitrite import [72]. NarG shares homology with NxrA nitrite oxidoreductase (EC 1.7.5.1) [20, 73, 74], which can also serve as either a nitrate oxidase or a nitrate reductase [74]. NxrA is more frequently encoded by *Thermodesulfovibrionia* (notably *Magnetobacteriaceae* and *Dissulfurispiraceae*) and by most *Nitrospiria* (Fig. S2, Table S7) [20]. No *napAB* periplasmic nitrate reductase genes were identified in class 9FT-COMBO-42-15 genomes, but they are common in *Thermodesulfovibrionia*. The nitrite product of NarGH could be further utilized by nzgw271, and other members of the genus, for respiration (via DNRA or the denitrification pathway) or for assimilation or fermentation via the ammonia-forming catalytic nitrite reductase subunit *nirB* [75] (*nirD* was universally absent [21]).

Ammonia-forming dissimilatory nitrite reductase genes, *nrfAH*, were present in MAG nzgw271, and two other species in the genus 9FT-COMBO-42-15, demonstrating the capacity to generate energy via the six-electron reduction of nitrite to ammonium [76]. DNRA prevails over denitrification under nitrate-limiting conditions [77], under more reducing conditions where electron acceptors are scarcer due to a greater consumption of electrons [78], and where carbon/nitrogen ratios are high [77]. In comparison, groundwater where nzgw271 was most abundant and transcriptionally active (sample gwj15) had mixed redox conditions (DO 0.37 mg/L) and high nitrate concentrations (4.4 g/m^3^) compared to nitrite (0.031 g/m^3^), which suggests a lack of resource limitation for denitrification (Fig. 3A). Although DOC was relatively high for groundwater (10.1 g/m^3^) [6], the C/N ratio (2:3) was much lower than previously shown to favour DNRA [79]. Accordingly, analysis of transcripts indicates nzgw271 was more likely engaged in denitrification at the time of sampling (Fig. 3C). Transcripts for the key step in DNRA (*nrfAH*) were not detected, unlike those for nitrate reduction (*narGH*) and steps specific to the denitrification pathway (*nirK*, *nirS*, *norC*, *nosZ*-like). Analysis of the overall prokaryotic community likewise indicated that gene transcription associated with denitrification was one-to-two orders of magnitude greater than DNRA [3].

#### Role in groundwater denitrification: capacity for steps from nitrate reduction to nitrous oxide generation

**Figure 4:**
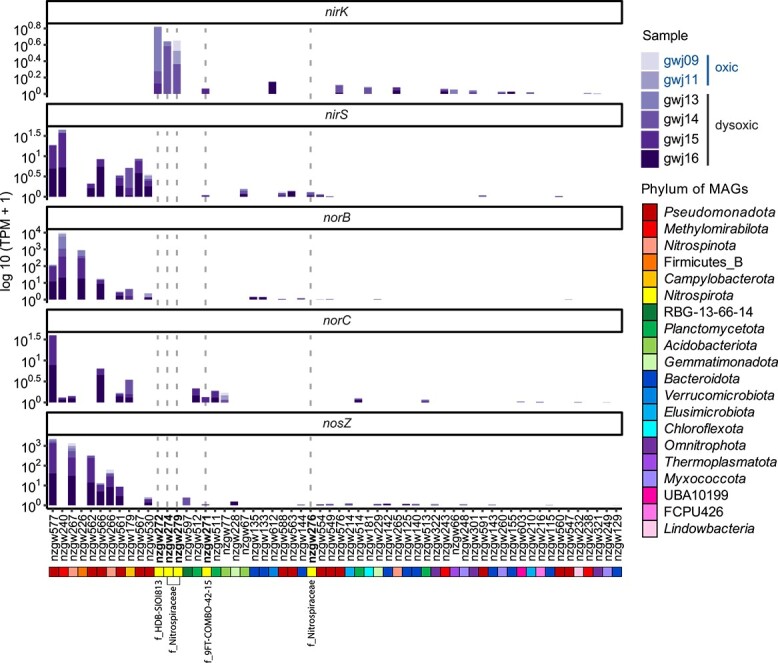
Activity of the denitrifying microbial community (TPM) across sites C (gwj09, gwj11) and D (gwj13–gwj16). Gene transcripts are shown for nitrite reductases (*nirK* and *nirS*), nitric oxide reductase (*norBC*), and nitrous oxide reductase (*nosZ*) for each MAG in which these genes were detected. MAG identifiers are shown along the *x*-axis. Bolded font and dashed grey lines indicate *Nitrospiria* MAGs.

The most transcriptionally active prokaryotes associated with denitrification in the sampled groundwater were from phyla, such as *Pseudomonadota*, *Methylomirabilota*, and *Nitrospinota*, based on summed *nir*, *nor*, and *nos* transcripts (TPM, Fig. 4). Nonetheless, denitrification tends to be a community effort [3, 62], and *Nitrospirota* from class 9FT-COMBO-4215 and *Nitrospiraceae* were among the more transcriptionally active contributors overall (five MAGs). This included nzgw271 (family 9FT-COMBO-4215) and nzgw272 (family HDB-SIOI813), which ranked 17th and 12th out of 60 taxa, respectively. Notably, all these *Nitrospirota* expressed the *nir* genes for converting nitrite into nitric oxide, particularly in dysoxic groundwater, and made the biggest transcriptional contribution to this step in the pathway (Fig. 4).

*Nir* comprises two structurally different, but functionally equivalent enzymes, cytochrome *cd*_1_-type nitrite reductase NirS, and copper-containing nitrite reductase NirK [62]. Most *Nitrospiria* and *Leptospirilla* contain *nirK*. Other classes contain an equal mix (9FT-COMBO-42-15), a few of which are mostly *nirS* (*Thermodesulfovibrionia*), or neither (UBA9217) (Fig. 1A and B). 9FT-COMBO-42-15 bacterium nzgw271 possesses both genes (Fig. 1A) and expresses both concomitantly in dysoxic groundwater (Fig. 3C). Oligotrophic strains of *Bradyrhizobia* also encode both nitrite reductases, and use NirS to facilitate swimming motility in the presence of nitrate [80]. Swimming motility would be advantageous for nutrient acquisition [81] in oligotrophic groundwater and in unconfined aquifers with fluctuating oxygen levels [82]. Members of the 9FT-COMBO-42-15 family largely lack genes for flagellar synthesis (an exception being the biosynthesis protein gene *flgG*), but most members of the HDB-SIOI813 family encode numerous synthesis genes, and likely undertake swimming motility (28–40 genes annotated in each of nine genomes, Table S8). Of these, members of genus HDB-SIOI813 encode flagellar (all five) and NirS (four of five) (Fig. 1B, Table S8), potentially providing a secondary role for NirS.

Nitric oxide is reduced to nitrous oxide by NorB (a heme-rich subunit containing the active site) and NorC (cytochrome *c*) [83]. NorBC genes are prevalent in *Thermodesulfovibrionia* and UBA9217, and *norBCDQ* was identified in one member of the HDB-SIOI813 genus. In contrast, nzgw271 and other members of the 9FT-COMBO-42-15 family are devoid of *norB*, although *norD* and *norQ* genes, essential for the activation of NorBC [83], are universally present [1]. The *norC* gene of nzgw271 was, nonetheless, transcribed in dysoxic groundwater alongside genes encoding nitrate and nitrite reductases, and a putative nitrous oxide reductase (Fig. 3C). Members of the 9FT-COMBO-42-15 family could therefore use a novel mechanism for NO reduction. Alternatively, other bacteria could complete this step [62], as illustrated by diverse taxa transcribing *norBC* in dysoxic groundwater (Fig. 4).

#### Capacity for the final step of denitrification

Results show diverse *Nitrospirota* could contribute to N_2_O conversion to N_2_ in aquifers (Fig. 1A) via NosZ with predominantly Tat-type signal peptides (clade I NosZ proteins; Table S9). Canonical nitrous oxide reductase, NosZ, proteins comprise two clades (I and II) that have different secretion pathways for delivering the protein across the cytoplasmic membrane [84, 85]. Clade I NosZ possess the twin-arginine translocation (Tat) signal peptide [86], whereas clade II NosZ (except *Chloroflexi nos*), possess an N-terminal Sec-type signal peptide [84]. *Nitrospirota* genes encoding the Tat-type NosZ are prevalent among *Thermodesulfovibrionia*, and are found among *Thermodesulfovibrionia* and RBG-16-62-22 sourced from groundwater (e.g. RBG-16-64-22 sp001803795) (Fig. 1A). Phylogenetic analysis shows that these Tat-type *Nitrospirota* NosZ protein sequences comprise a sub-clade within clade I, closely related to gammaproteobacterial NosZ, indicating a recent evolutionary divergence (Fig. S3).

**Figure 5:**
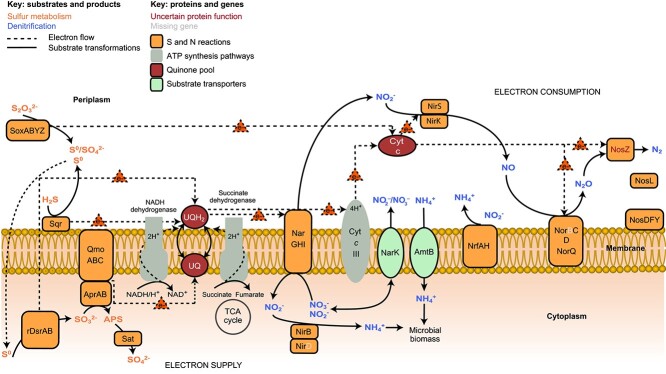
Schematic of autotrophic sulphur-dependant denitrification pathways genomically inferred from MAG nzgw271. Orange font indicate substrates and products involved in S metabolism; blue indicates denitrification. Dashed lines = electron flow. Solid lines = substrate transformations. Red font denotes uncertain protein function. Pale grey font denotes missing genes. Shapes: Orange = proteins involved in sulphur and nitrogen reactions, grey = ATP synthesis pathways, brown = quinone pool, green = substrate transporters.

Proteins annotated as Sec-type NosZ are almost exclusively encoded by bacteria in class 9FT-COMBO-42-15 (Table S9). These proteins (including one from a *Nitrospiria* in the SBBL0 order) are phylogenetically distinct from canonical clade II Sec-types (Fig. S3). They form a distantly related clade shared by sequences from a diverse collection of other taxa—*Nitrospinota* bacterium “DRJW01 sp011052055” (GCA_011052055.1, derived from a hydrothermal vent) [87], *Sporomusa acidovorans* DSM 3132 (which also has genes for DNRA) [88], and a *Burkholderiales* species (GCA_905339285.1). N_2_O reductases are characterized by a 7-bladed β-propeller domain and cupredoxin domain [89]. The novel clade of NosZ-like proteins contains a cupredoxin domain. The 7-bladed beta-propeller domain is homologous to a lactonase (IPR019405, Fig. S4, Table S9) rather than the usual Nos β-propeller, explaining the sequence divergence. Inspection of MAG nzgw271 showed the presence of the novel *nosZ*-like gene in addition to *nosDFLYX* genes (Fig. 5), which are found in canonical *nos* clusters [84]. The *nosZ*-like gene was expressed in dysoxic groundwater along with other denitrification genes (Figs 3A and 4; Table S6). However, experimentation is needed to confirm if this protein performs nitrous oxide reduction or some other function.

#### Diverse mechanisms for sulphur oxidation

**Figure 6:**
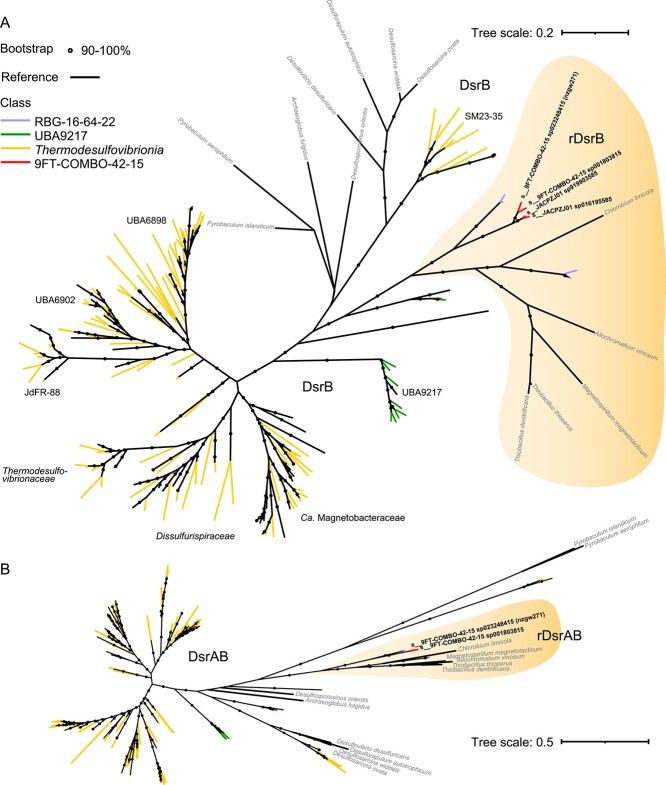
Maximum-likelihood consensus trees of Dsr protein sequences among *Nitrospirota*. The trees show the reverse/oxidative (rDsr) type in 9FT-COMBO-42-15 and RBG-16-64-22, and reductive type (Dsr) among UBA917 and *Thermodesulfovibrionia*. (A) DsrB only. Leaves were dispersed using the equal-daylight algorithm in iTOL. (B) Concatenated DsrAB alignments (where r/DsrA and r/DsrB were on the same contig). Leaves are undispersed. Leaf labels in bold black font indicate 9FT-COMBO-42-15 genomes, and grey font indicates reference taxa for *dsrAB* (e.g. *Desulfovibrio desulfuricans* and *Archaeoglobus fulgidus*) and r*dsrAB* (e.g. *Thiobacillus denitrificans* and *Chlorobium limicola*) genes. Clade labels in unbolded black font show the dominant family. Trees are unrooted, and were constructed using model LG + F + I + G4, and with 1000 ultrafast bootstrap replicates.

Mechanisms for sulphur cycling vary across *Nitrospirota* clades (Dsr, rDsr, Sox, Hdr/Qmo, Sqr), including lineages over-represented in aquifers (Fig. 2A). Analyses showed that dissimilatory sulphite reductase genes (*dsrAB*), indicative of sulphate reduction, are pervasive across the basal *Thermodesulfovibrionia* and UBA9217 classes (Figs 1A and 6) [20], and in numerous aquifer-derived members of these classes (Fig. 1A). The capacity for sulphate reduction has previously been shown for *Thermodesulfovibrio* species in culture [27] and predicted for *Candidatus* Magnetobacterium species [68]. A sulphate-reducing metabolism is also consistent with the high relative abundance of *Thermodesulfovibrionia* in suboxic-to-anoxic groundwaters sampled (Fig. 2A) and the widespread availability of sulphate [3]. Phylogenetic analyses indicate these “reductive” type *dsrAB* genes are lacking from the other *Nitrospirota* classes (Fig. 6). In contrast, DsrAB protein sequences encoded by nzgw271 and other members of family 9FT-COMBO-42-15, along with RBG-16-64-22, are the reverse or “oxidative” type (Fig. 6), and as predicted for 9FT-COMBO-42-15 H51-bin250-1 [21] (nzgw271 and H51-bin250-1 DsrAB protein-coding sequences shared 92.2% and 94.7% identity). All members lack *dsrD* genes (Fig. 1A), which are typical of sulphate-reducers or sulphur-disproportionating organisms, and MAGs nzgw271 and H51-bin250-1 (and RBG-16-64-22 genomes) instead possess *dsrEFH*, which are considered unique to sulphur-oxidizers [90]. Although it should be noted that most *Thermodesulfovibrionia* with only reductive-type *dsrAB* genes also lack *dsrD*, and possess *dsrFH*, but no *dsrE*. A marked exception is the *Thermodesulfovibrionia* family SM25-35, for which multiple members possess *dsrD* and lack all *dsrEFH* genes. The *dsrAB*, r*dsrAB*, and related genes in *Nitrospirota* were probably acquired through a series of lateral gene transfer events [22]. There was no evidence of these genes being carried by a genomic island in nzgw271, although other transfer mechanisms cannot be discounted.

The capacity for sulphur oxidation strongly differentiated the two 9FT-COMBO-42-15 families. Little evidence was found for sulphur oxidation (except via Sqr or potentially Hdr/Qmo) in the HDB-SIOIB13 family, whereas all members of the 9FT-COMBO-42-15 family possessed genes associated with reverse Dsr and the sulphur oxidation pathway (Sox), which were most complete for genus 9FT-COMBO-42-15 (Fig. 1A). Strikingly, one member of the 9FT-COMBO-42-15 genus (MAG nzgw271) possessed a full complement (*soxXAYZB* and r*dsrABCEFHL*), excluding *soxCD*, in addition to Sqr and Hdr/Qmo genes (Fig. 1B; Table S6), which suggests it can oxidize a variety of sulphur species (sulphide, elemental sulphur, sulphite, thiosulphate) to sulphate (Fig. 5), and that sulphur represents an important energy source. The sulphide:quinone oxidoreductase (Sqr) predicted is involved in poly-S formation and catalyzes the oxidation of sulphide to elementary sulphur for respiration and potentially also detoxification [91]. Heterodisulphide reductase genes (*hdrABC/qmoABC*), present in nzgw271, could be used to oxidize sulphur compounds to sulphite [22]. Sulphite could then be oxidized to sulphate by genes encoding adenosine 5′-phosphosulphate reductase (*aprAB*) and adenosine 5′-triphosphate (ATP) sulfurylase (*sat*) (Figs 1B and 5) [22]. Alternatively, elementary sulphur could be oxidized to sulphite by the dissimilatory sulphite reductase encoded by r*dsrABCEFHL* genes [22].

Thiosulphate is a product of sulphur-reducing bacteria or is formed through the chemical oxidation of hydrogen sulphide [92]. Thiosulphate oxidation could be catalyzed by the sulphur oxidation multienzyme complex (*soxXAYZB*) [93]. MAG nzgw271 was devoid of genes encoding sulphur dehydrogenase SoxCD, and they were likewise entirely missing from all other early branching/basal clade genomes, including early branching *Nitrospiria* (Fig. 1A) [20]. This is consistent with *soxCD* absence in other bacteria encoding r*dsrAB* [92]. In *Paracoccus pantotrophus* (the model organism for Sox), SoxCD facilitates growth on thiosulphate by oxidizing protein-bound sulphur (sulfane to sulfone) and increasing the electron yield from two to eight [93]. However, rDsr is proposed to substitute for SoxCD in green sulphur bacteria to enable complete oxidation of thiosulphate to sulphate [92, 94], and may be likewise utilized by nzgw271 and other similarly equipped *Nitrospirota*. Sox systems devoid of SoxCD are found in a broad range of sulphur-oxidizers. Genes encoding SoxCD are missing from *Thiobacillus denitrificans* and *Thioalkalivibrio* HL-EbGR7, which are capable of sulphur-driven autotrophic denitrification [4], and subsurface-derived *Sulfuricurvum* species [95, 96]. *Sulfuricurvum kujiense* oxidizes various sulphur species aerobically/anaerobically, including coupling nitrate reduction with thiosulphate-oxidation [95]. The Sox pathway sulphur dehydrogenase genes were instead exclusively found among the *Nitrospirales* (*Nitrospiria*) (Fig. 1A). Although SoxCD in *P. pantotrophus* was shown to have no catalytic activity in the absence of other sox proteins [93], *soxCD* genes are prevalent among *Nitrospirales* [20] (Fig. 1A). *Nitrospirales* are characterized by genes for nitrification (*nxrAB* + *amoABC*) and overwhelmingly lack other *sox* genes (Fig. 1A). They possibly use reduced forms of sulphur (via SoxCD or Sqr) as an alternative electron sink or for detoxification [91].

#### Coupled sulphur and nitrogen metabolism

Reduced sulphur compounds, such as sulphide, elemental sulphur, and thiosulphate, can act as electron donors when coupled to the reduction of nitrate, leading to partial or complete denitrification and the formation of oxidized sulphur species, such as sulphate [95]. As nzgw271 encodes nitrate reductase and multiple nitrite reductases (NirS, NirK, NrfA) and mechanisms for sulphur oxidation (rDsr, Sox, Sqr, Hdr/Qmo), it could flexibly couple either of two respiratory nitrate/nitrite reduction pathways (denitrification or DNRA) with sulphur oxidation (Fig. 5). Co-expression of denitrification genes (*narGH*, *nirS*/*nirK*, *norC*, *nosDLZ*) with *dsrA* and *qmoC* genes additionally suggests nzgw271 may have actively coupled sulphur oxidation to denitrification at the time of sampling (Fig. 1C). Simultaneous expression of *hdr/qmo* genes with those for denitrification has been observed in *Thiobacillus* and *Thauera* [97], indicating a role for *hdr/qmo* in sulphur-based denitrification. Similar N and S cycling mechanisms in the 9FT-COMBO-42-15 family (e.g. nzgw271) and class RBG_16_64_22 suggest that phylogenetically disparate *Nitrospirota* in aquifers are capable of sulphur-driven denitrification (Fig. 1, Supplementary Information).

## Conclusion

Results show that phylogenetically and metabolically diverse *Nitrospirota* genera are present in aquifers, spanning all seven known classes. However, certain lineages are almost entirely comprised of members from aquifers (three clusters of *Thermodesulfovibrionia*, classes UBA9217, RBG-16-64-22, and 9FT-COMBO-42-15, and very early branching *Nitropiria*). In aquifers, *Nitrospirota* community fractions are structured based on groundwater oxygen contents ranging from oxic (*Nitrospira-*dominated with *Leptospirillum*) to anoxic (*Thermodesulfovibrionia*-dominated with other early branching clades, including *Nitrospiria*). This distribution reflects the distinct metabolic attributes of these lineages, in particular those characterized by aerobic nitrite oxidation versus nitrate reduction and sulphur oxidation or reduction. Most *Nitrospirota* genomes recovered from aquifers to date exhibit features associated with anaerobic nitrogen- and sulphur-based metabolisms and utilization of the WL pathway, rather than the rTCA cycles encoded by *Nitrospiria* and *Leptospirillum*. Genomic analysis of the aquifer-associated and recently established 9FT-COMBO-42-15 class indicates a transition among the two constituent families in their mechanisms for both sulphur metabolism (extensive in the 9FT-COMBO-42-15 family) and carbon acquisition (WL versus rTCA mediated). The transition reflects the intermediate phylogenetic placement of this class, and attributes consistent with basal clades or the later-evolved *Nitrospiria* class, respectively. Analysis of the 9FT-COMBO-42-15 family demonstrates its capacity for versatile autotrophic sulphur and nitrogen-based metabolisms, including DNRA and the denitrification pathway coupled to sulphur oxidation, which likely confers a broad niche breadth to this group of *Nitrospirota* in aquifers. The predicted coupling of nitrogen and sulphur metabolisms is supported by associated transcriptional activity in dysoxic groundwater indicative of sulphide oxidation and denitrification (alongside carbon fixation), although the capacity to undertake the complete denitrification pathway remains to be determined. Overall, results illustrate that metabolic adaptations among *Nitrospirota*, associated with the capacity to undertake oxidative or reductive transformations of nitrogen and sulphur species, enable members of this phylum to colonize geochemically distinct groundwaters and that these adaptations contribute to the spatial differentiation of lineages in terrestrial aquifers.

## Supplementary Material

Supplementary-Information-Mosley-2024_ycae047

Supplementary-Tables-Mosley-2024_ycae047

## Data Availability

All metagenomic, metatranscriptomic and amplicon sequence data are available from National Center for Biotechnology Information (NCBI) under BioProject PRJNA699054.
