# Supplementary material for: Implications for nitrogen and sulphur cycles: phylogeny and niche-range of Nitrospirota in terrestrial aquifers
Source: ISME Commun. 2024 Mar 27;4(1):ycae047. doi: 10.1093/ismeco/ycae047 (PMC11033732; doi:10.1093/ismeco/ycae047)
Supplement: Supplementary-Information-Mosley-2024_ycae047 [file supplementary-information-mosley-2024_ycae047.pdf]

Supplementary Information (Mosley *et al.*)

MATERIALS AND METHODS

Metatranscriptome analysis

Adapter removal from transcriptomic reads and quality trimming was as for metagenomic reads. Residual ribosomal RNA sequences were removed using SortMeRNA v2.1 [1], and checked to ensure filtered reads were still paired using BBMap v38.81 “repair.sh” [2]. Filtered transcriptomic reads were mapped to dereplicated MAGs using Bowtie2 v2.3.5 [3] (--end-to-end --very_sensitive). Read counts were determined using featureCounts v1.6.3 [4] (-F SAF). Read counts were normalised to a modified version of transcripts per kilobase per million reads mapped (TPM) [5] using the following equation: (number of reads mapped to gene)*(1000/gene length)*(1000000/library size) and singleton mapped reads per gene were removed before analysis.

Genome coverage and taxonomy

Adapters were removed from metagenomic reads using cutadapt v2.10 [6], and reads were then trimmed and quality checked using sickle [7] (Phred score ≥ 30; read length ≥ 80 bp) and FastQC v0.11.7 [8], respectively. For genome coverage, trimmed reads were mapped onto dereplicated genomes using Bowtie2 v2.3.2 [3] (-n 1 -l 222 --minins 200 --maxins 800 –best). Sample-specific genome relative abundance was calculated by normalising to library size and highest read count [9].

RESULTS AND DISCUSSION

Carbon fixation

Carbon fixation pathways: Reductive acetyl-CoA (Wood-Ljungdahl) pathway. The capacity for carbon dioxide fixation via the reductive acetyl-CoA (Wood-Ljungdahl) pathway was recently found in *Nitrospirota* species, such as *Ca.* Magnetobacterium [10]. The capacity is prevalent among earlier *Nitrospirota* based on the near ubiquitously identified carbon monoxide dehydrogenase *codhA*/*acsA* and *codhC*/*acsB* genes (Fig. 2a) [11]. In nzgw271, key enzymes for both the western (carbonyl) and eastern (methyl) branches were found. The western branch incorporates a carbon dioxide molecule into the carboxylic group of an acetate molecule [12, 13]. In nzgw271, western branch genes encoding carbon monoxide

dehydrogenase (*codhA/acsA*) and acetyl-CoA synthase (*codhC/acsB*) were present and expressed (Fig. 3), suggesting an autotrophic based metabolism [14]. Western branch genes were also present that encode the acetyl-CoA synthase corrinoid iron–sulfur protein (*codhE/acsC*) and the anaerobic carbon-monoxide dehydrogenase iron sulfur subunit (*cooF*). The *codhD/acsD* gene was absent from the nzgw271 MAG, but present in other members of the genus and family.

Genes comprising the five steps of the eastern branch were also present in MAG nzgw271. The encoded NADP-dependent formate dehydrogenase (FdhAB), catalyses the reversible conversion of carbon dioxide to formate [15] (step 1), and formate-tetrahydrofolate (THF) ligase (Fhs) generates formyl-THF (step 2). The encoded bifunctional methenyltetrahydrofolate cyclohydrolase and methylenetetrahydrofolate dehydrogenase (NADP+), FolD, catalyses steps 3-4: formyl-THF → 5,10-methenyl-THF, and then 5,10-methenyl-THF → 5,10-methylene-THF. Conversion of 5,10-methylene-THF to 5-methyl-THF (step 5) is achieved via methylene tetrahydrofolate reductase, and could be undertaken by nzgw371 using the encoded MetF large subunit and co-located methylenetetrahydrofolate reductase C-terminal domain-containing protein. The product could then be converted by the encoded western branch methyltetrahydrofolate:corrinoid/iron-sulfur protein (AcsE) into methyl-Co(III) corrinoid Fe-S and combined with CO (by the encoded AcsB) (Fig. 2b).

This pathway has been best studied in homoacetogenic bacteria, including *Sporomusa* species [16], that synthesize acetate from H_2 and CO_2 [17]. Some sulfate-reducing organisms and aceticlastic methanogens can also run the Wood-Ljungdahl in reverse, under anoxic conditions [12, 18], utilizing the eight electrons generated during acetate oxidation to reduce sulfate to H_2S (using western and eastern branches) [19]. *Ca.* Magnetobacterium is predicted to exhibit this trait under sulfate-reducing conditions [10], which may also be undertaken by other basal *Nitrospirota* clades (Fig. 2a).

Tricarboxylic acid (TCA) cycle and RubisCO. Most members of the *Nitrospiria* encode a complete reductive tricarboxylic acid (rTCA) cycle for inorganic carbon fixation, which is suggested to have evolved from microaerophilic or anaerobic ancestors [20], similar to the predicted lifestyles of other *Nitrospirota* in this study. MAG nzgw271 contains an incomplete rTCA pathway, as previously identified in obligate autotrophs and methanotrophs [21]. The acetyl-CoA entering this pathway may be biosynthesized by the acetyl-CoA pathway, or from acetate by acetyl-CoA synthetase (*acs*). Key enyzmes for the rTCA pathway, such as pyruvate:ferredoxin oxidoreductase (*porABCD*; PFOR), pyruvate carboxylase (*pycAB*), and 2-oxoglutarate:ferredoxin oxidoreductase (*korABCD*; OGOR) (*korB* was actively expressed), are present (Fig. 2b), and are predicted to incorporate carbon dioxide during central carbon

metabolism [22]. ATP citrate synthase (*aclA*) and citrate synthase (*gltA*) were absent, however the genome encodes a citrate *Re*-synthase which converts acetyl-CoA and oxaloacetate to citrate [23] in the TCA cycle. The citrate *Re*-synthase had 54% similarity to the protein in *Clostridium klyuveri*, which is inactivated by oxygen [23].

MAG nzgw271 also has several genes encoding the second carbon oxidation pathway (2-oxoglutarate → oxaloacetate) of the TCA cycle: 2-oxoglutarate:ferredoxin oxidoreductase (*korABCD*), succinate dehydrogenase/fumarate reductase (*sdhABCD*), fumarate hydratase (*fumAB*) and malate dehydrogenase (*mdh*), although genes encoding the reversible reaction of succinate to succinyl-CoA, *sucCD,* LSC1, LSC2, were absent). Incomplete TCA and rTCA cycles can still convert pyruvate to necessary biosynthetic intermediates under anaerobic, or microaerophilic conditions, and is suggested to be from an intermediate stage in the evolution of the oxidative catabolic TCA cycle that was primarily for amino acid biosynthesis, catalysed by a non-cyclic "horseshoe" pathway that lacks an enzymatic step to form succinate [21].

Additionally, ribulose-bisphosphate carboxylase (*cbbL*) from the ribulose-1,5-bisphosphate carboxylase/oxygenase (RubisCO) pathway is present in the nzgw271 genome. It was most similar to the protein-coding sequence of a *Nitrospirae* bacterium (87.6% identity to GCA_016214425.1, NCBI) and RuBisCO-like protein in *Rhodospirillum rubrum*. The latter belongs to the *t*ype IV RuBisCO subfamily (37% identity). *Ca.* Magnetobacterium bavaricum [24] also contains type IV RubisCo-like proteins (41.7% identical to nzgw271), further verified in *Ca.* Magnetobacterium caviensis [10]. Type IV forms lack RubisCO activity and are suggested to be involved in thiosulfate oxidation rather than CO_2 fixation in *Ca.* Magnetobacterium bavaricum [24]. It is also mostly likely that that the Calvin–Benson–Bassham pathway for carbon dioxide fixation is not operational in nzgw271 due to a lack of fructose-bisphosphate aldolase, fructose-bisphosphatase, sedoheptulose-bisphosphatase and phosphoribulokinase genes.

Genomic flexibility

Several respiratory chain complexes, which are used for energy generation and electron transport are encoded by nzgw271, such as NADH:quinone oxidoreductase, succinate dehydrogenase and cytochrome *c* oxidase (≥ 50% complete) [25]. One gene encoding the large subunit of unidirectional NiFe Group 1b-hydrogenase for hydrogenotrophic respiration is also present. This hydrogenase uses sulfate, fumarate, nitrate, metals and azo compounds as terminal electron acceptors and is generally found in anoxic environments [26]. MAG

nzgw271 may therefore grow anaerobically by nitrate respiration using hydrogen as an electron donor, as for *Wolinella succinogenes* [27]. Comparably, *Nitrospira* can oxidise hydrogen in place of nitrite [28, 29]. Results therefore suggest the genome flexibility exhibited by *Nitrospira* may also be exhibited by 9FT-COMBO-42-15 bacterium nzgw271. This flexibility may enable nzgw271 to optimize its energy metabolism to local geochemical conditions.

Coupled sulfur and nitrogen metabolism

The potential for coupling sulfur and nitrogen metabolism could be present in other physiologically diverse *Nitrospirota*. The capacity for nitrate-dependent sulfur oxidation (e.g., via Sqr) has also been predicted for *Ca.* Magnetobacterium [10, 24], despite possessing reductive-type *dsrAB* genes (Fig. 6, Table S3). The same capacity could be harboured by scattered *sox*-bearing groups of *Thermodesulforovibronia* (Fig. 2a). However, as these *sox*-bearing groups encode reductive-type Dsr genes (as for most *Thermodesulforovibronia*) it is possible that they do not conserve energy for growth via Sox-mediated sulfur oxidation [30]. Moreover, some N-cycling genes in these organisms (e.g. *nrfAH*) could serve primarily to detoxify inhibiting nitrite during sulfate reduction [31].

Supplementary Figures

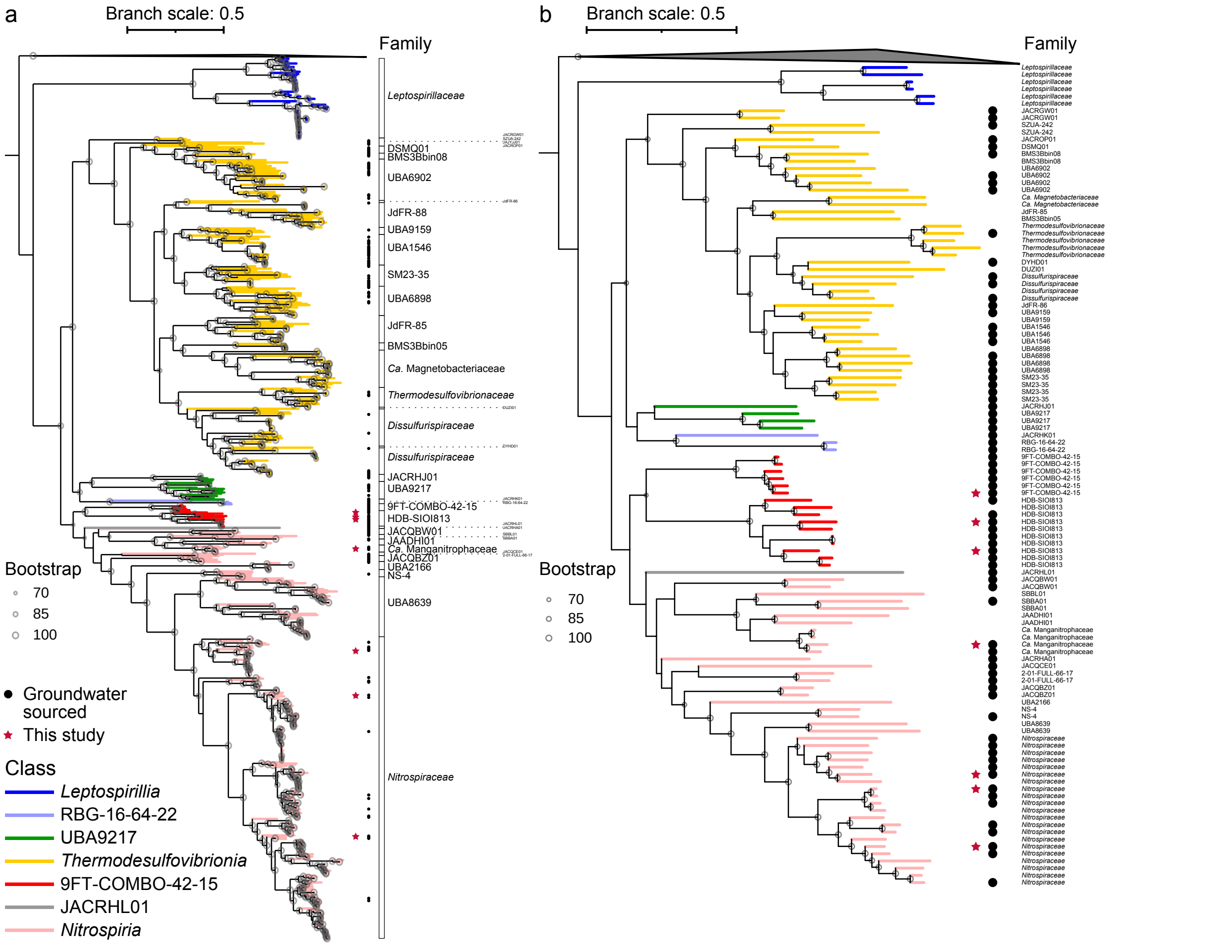

Figure S1. Phylogenomic trees of *Nirospirota* genomes based on concatenated core gene protein sequence alignments. (a) The phylogenetic association of 750 *Nitrospirota* genomes, as in figure 2, with ultrafast bootstrap values indicated (1,000 replicates). (b) A similar tree topology obtained with a subset of 115 *Nitrospirota* genomes and 1,000 standard bootstrap replicates. The tree includes representatives of all seven classes and 19 orders in the *Nitrospirota*, and at least one family representative of genomes from terrestrial aquifer sources (30 out of 42 *Nitrospirota* families). Branches in both trees are coloured by class. Branch labels: red stars = genomes from this study; black circles: genomes from terrestrial aquifers; text = family designations. Trees are rooted to an outgroup comprising 11 other phyla (grey-shaded collapsed clades) given in Table S1. Placement of the single representative of JACRHL01 was poorly supported (nominal GTDB phylum designation Nitrospirota_B). *Leptospirillum* (nominal GTDB phylum designation Nitrospirota_A) was placed as a distinct clade, although 16S rRNA gene based alignments have placed *Leptospirillia* as a sister clade of *Nitrospiria* (e.g., SILVA SSU Ref NR 99 guide tree [32]) or within *Nitrospiria* [33, 34].

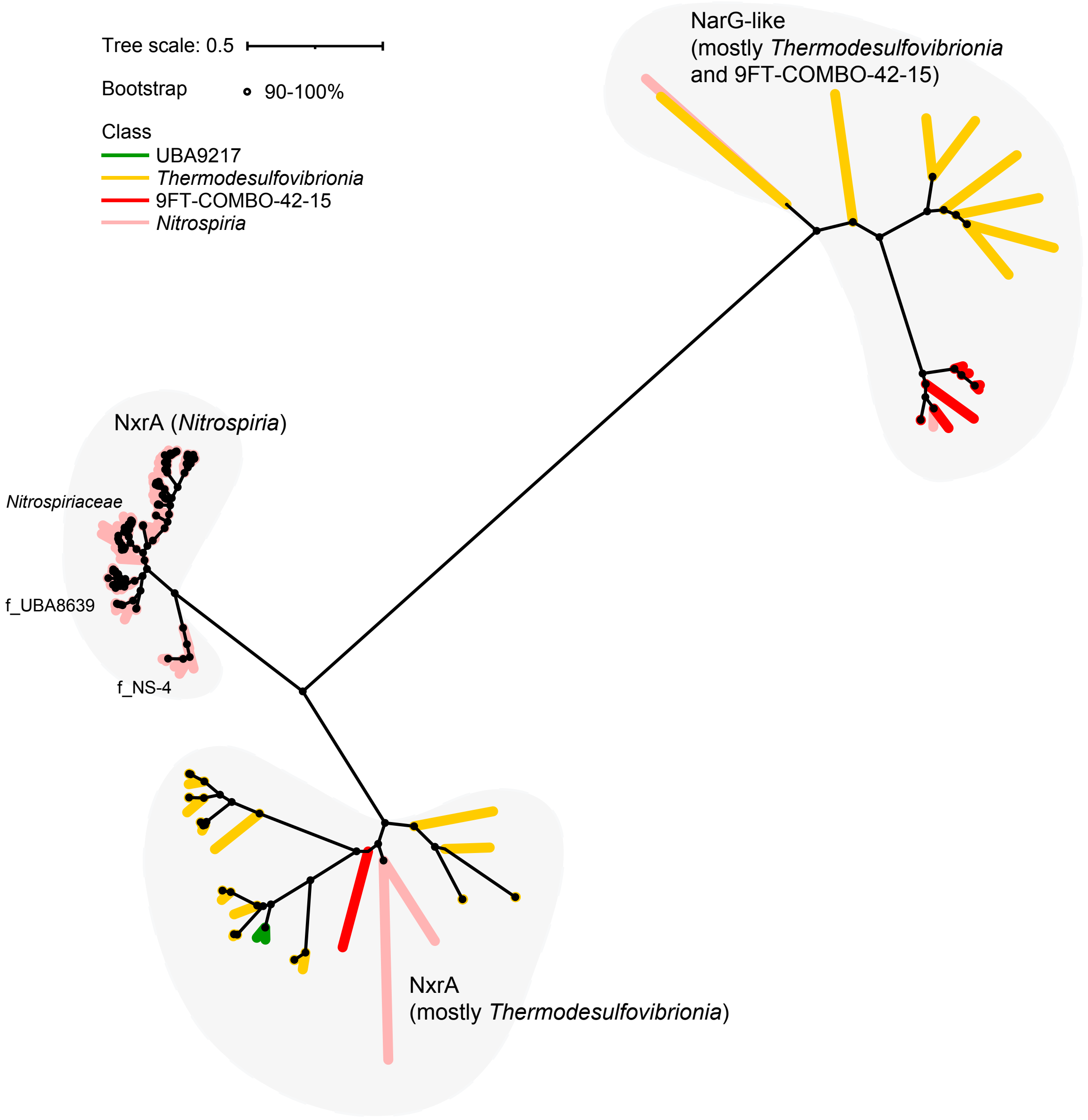

Figure S2. Maximum-Likelihood consensus tree of NxrA and NarG proteins in *Nitrospirota*. Leaves were dispersed using the equal-daylight algorithm in iTOL. The tree is unrooted, and was constructed using model LG+F+I+G4, and with 1,000 ultrafast bootstrap replicates.

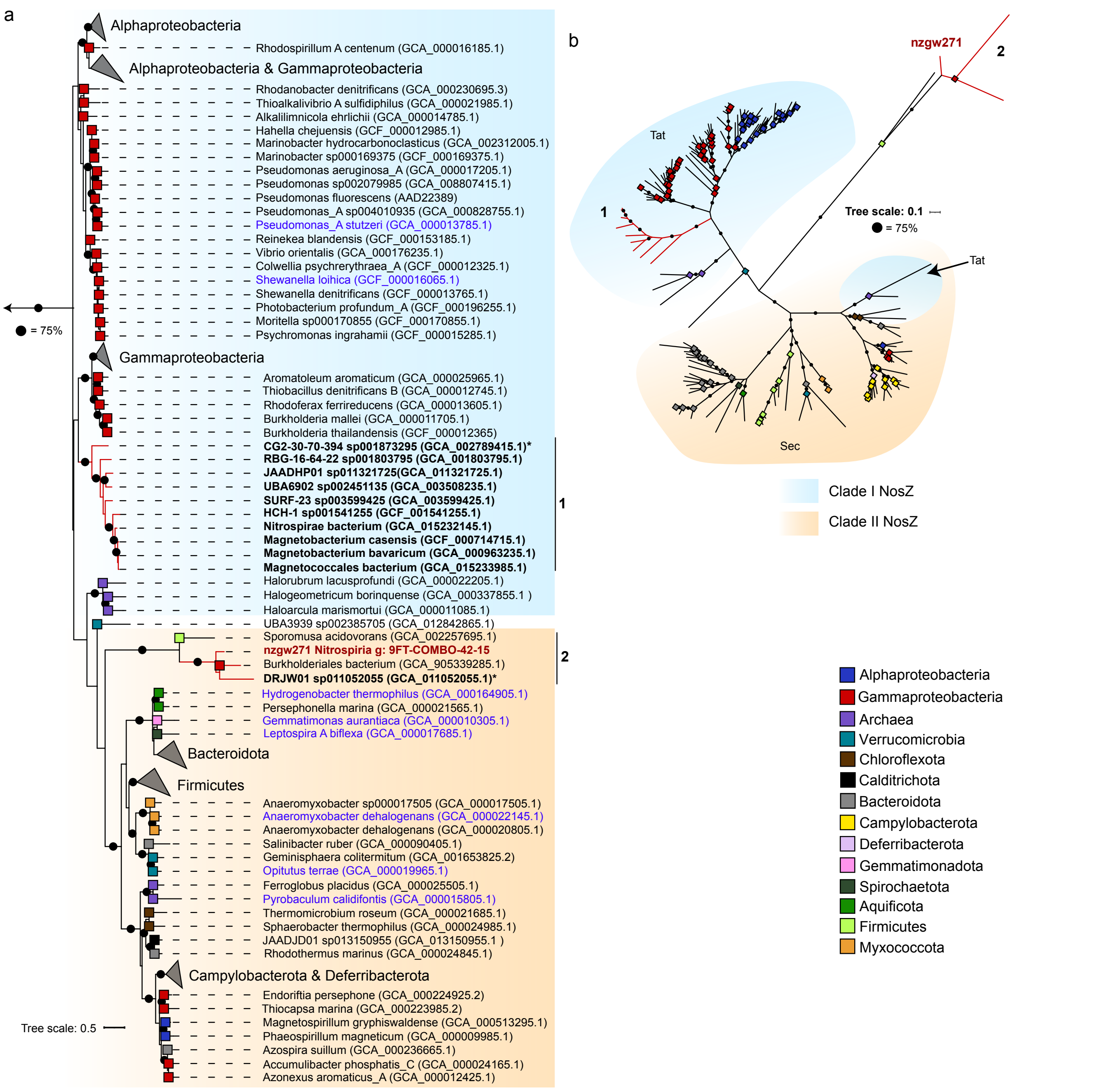

Figure S3. Phylogenetic tree of nitrous oxide reductase (NosZ) protein subunits from *Nitrospirota* along with other phyla. (a) Rooted tree showing phyla (coloured square). Sequences are labelled to lowest level of taxonomy (GTDB) with accession number. * indicates MAGs classified as *Nitrospirae* (NCBI), but are non-*Nitrospirota* (GTDB). (b) Unrooted phylogenetic tree with identical NosZ sequences from (a). Red branches indicate Nitrospirota (GTDB)/Nitrospirae (NCBI) genomes, which form 2 distinct clusters, labelled 1 (part of NosZ clade I) and 2 (comprising a putative NosZ clade III). The arrow highlights a subset of taxa that encode Tat-based clade I NosZ, but are phylogenetically more closely related to taxa encoding Sec-based clade II NosZ. Red font indicates MAG nzgw271 from this study. Blue font indicates confirmed nitrous oxide reducers/denitrifers. Each tree was made using LG+F+I+G4 model with 1,000 bootstraps. Black circles on tree branches represent ≥ 75% bootstrap value. Scale bars represent number of substitutions per site.

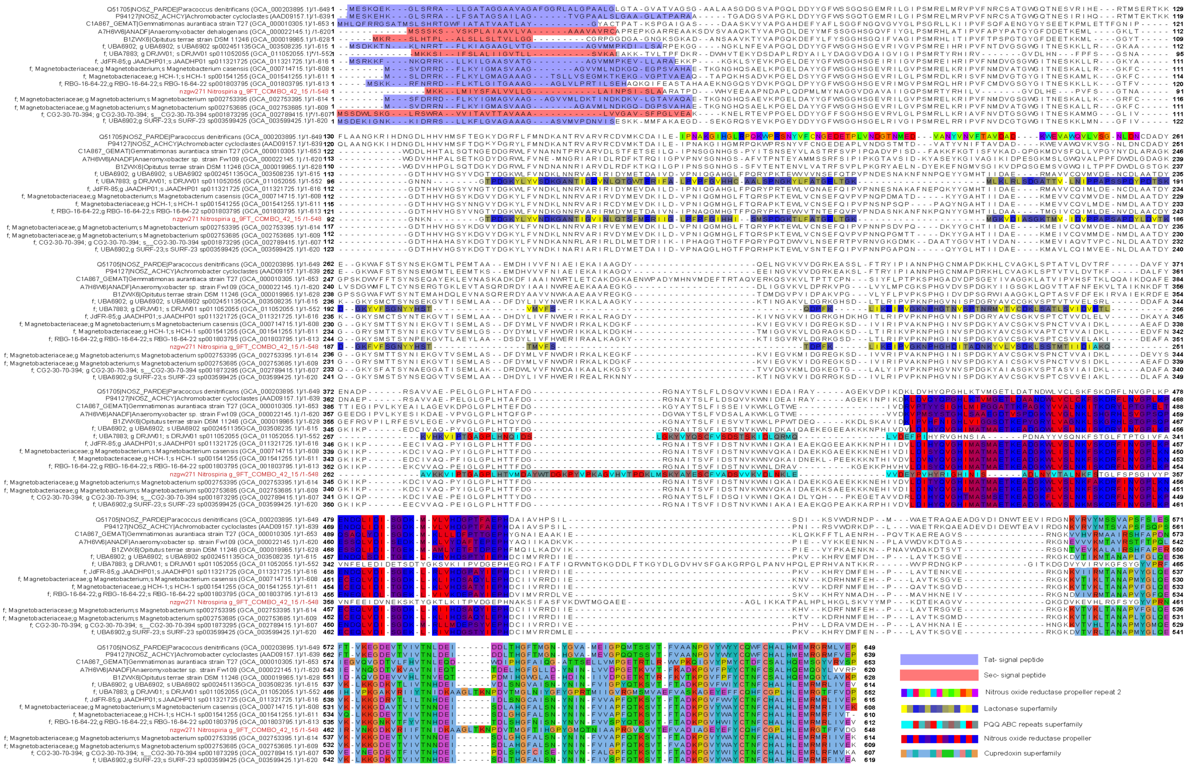

Figure S4. Amino acid alignments (generated using MUSCLE, default parameters) of pre-trimmed NosZ proteins. Signal peptides and protein domains of characterised nitrous oxide reducers, and the lactonase-domain encoding sequence in *Nitrospirota* 9fT-COMBO-42-15 MAG nzgw271 and *Nitrospinota* f_UBA7883, were annotated with JalView v2.11.14 [35].

REFERENCES

1. Kopylova E, Noé L, Touzet H. SortMeRNA: fast and accurate filtering of ribosomal RNAs in metatranscriptomic data. *Bioinformatics*. 2012; **28:** 3211-3217.
2. Bushnell B. BBMap: A fast, accurate, splice-aware aligner. Lawrence Berkeley National Laboratory, Berkeley, CA, United States, 2014.
3. Langmead B, Salzberg SL. Fast gapped-read alignment with Bowtie 2. *Nat Methods*. 2012; **9:** 357-359.
4. Liao Y, Smyth GK, Shi W. featureCounts: an efficient general purpose program for assigning sequence reads to genomic features. *Bioinformatics*. 2014; **30:** 923-930.
5. Wagner GP, Kin K, Lynch VJ. Measurement of mRNA abundance using RNA-seq data: RPKM measure is inconsistent among samples. *Theory Biosci*. 2012; **131:** 281-285.
6. Martin M. Cutadapt removes adapter sequences from high-throughput sequencing reads. *EMBnet J*. 2011; **17:** 10.14806/ej.17.1.200.
7. Joshi N, Fass J. Sickle: A sliding-window, adaptive, quality-based trimming tool for FastQ files, version 1.33. 2011. https://github.com/najoshi/sickle.
8. Andrews S. FastQC: A quality control tool for high throughput sequence data. 2010. http://www.bioinformatics.babraham.ac.uk/projects/fastqc/.
9. Probst AJ, Ladd B, Jarett JK, Geller-McGrath DE, Sieber CMK, Emerson JB et al. Differential depth distribution of microbial function and putative symbionts through sediment-hosted aquifers in the deep terrestrial subsurface. *Nat Microbiol*. 2018; **3:** 328-336.
10. Lin W, Deng A, Wang Z, Li Y, Wen T, Wu L-F et al. Genomic insights into the uncultured genus ‘Candidatus Magnetobacterium’ in the phylum Nitrospirae. *ISME J*. 2014; **8:** 2463-2477.

11. D'Angelo T, Goordial J, Lindsay MR, McGonigle J, Booker A, Moser D et al. Replicated life-history patterns and subsurface origins of the bacterial sister phyla Nitrospirota and Nitrospinota. *ISME J*. 2023; **17:** 891-902.
12. Hattori S, Galushko AS, Kamagata Y, Schink B. Operation of the CO dehydrogenase/acetyl coenzyme A pathway in both acetate oxidation and acetate formation by the syntrophically acetate-oxidizing bacterium *Thermacetogenium phaeum*. *J Bacteriol*. 2005; **187:** 3471-3476.
13. Schauder R, Preuß A, Jetten M, Fuchs G. Oxidative and reductive acetyl CoA/carbon monoxide dehydrogenase pathway in *Desulfobacterium autotrophicum*. *Arch Microbiol*. 1988; **151:** 84-89.
14. Ragsdale SW, Kumar M, Seravalli J, Qiu D, Spiro TG. Anaerobic Carbon Monoxide Dehydrogenase. In: Lidstrom ME, Tabita FR (eds). Microbial Growth on C1 Compounds. Springer Netherlands: Dordrecht, 1996. pp 191-196.
15. Ragsdale SW, Pierce E. Acetogenesis and the Wood-Ljungdahl pathway of CO(2) fixation. *Biochim Biophys Acta*. 2008; **1784:** 1873-1898.
16. Humphreys JR, Daniel R, Poehlein A. Genome sequence of the homoacetogenic, gram-negative, endospore-forming bacterium *Sporomusa acidovorans* DSM 3132. *Genome Announc*. 2017; **5:** e00981-00917.
17. Diekert G, Wohlfarth G. Metabolism of homocetogens. *Antonie Van Leeuwenhoek*. 1994; **66:** 209-221.
18. Jiao JY, Fu L, Hua ZS, Liu L, Salam N, Liu PF et al. Insight into the function and evolution of the Wood-Ljungdahl pathway in Actinobacteria. *ISME J*. 2021; **15:** 3005-3018.
19. Can M, Armstrong FA, Ragsdale SW. Structure, function, and mechanism of the nickel metalloenzymes, CO dehydrogenase, and acetyl-CoA synthase. *Chem Rev*. 2014; **114:** 4149-4174.

20. Lücker S, Wagner M, Maixner F, Pelletier E, Koch H, Vacherie B et al. A *Nitrospira* metagenome illuminates the physiology and evolution of globally important nitrite-oxidizing bacteria. *Proc Natl Acad Sci USA*. 2010; **107:** 13479-13484.
21. Wood AP, Aurikko JP, Kelly DP. A challenge for 21st century molecular biology and biochemistry: what are the causes of obligate autotrophy and methanotrophy? *FEMS Microbiol Rev*. 2004; **28:** 335-352.
22. Hügler M, Wirsen CO, Fuchs G, Taylor CD, Sievert SM. Evidence for autotrophic CO2 fixation via the reductive tricarboxylic acid cycle by members of the epsilon subdivision of proteobacteria. *J Bacteriol*. 2005; **187:** 3020-3027.
23. Li F, Hagemeier CH, Seedorf H, Gottschalk G, Thauer RK. Re-citrate synthase from *Clostridium kluyveri* is phylogenetically related to homocitrate synthase and isopropylmalate synthase rather than to Si-citrate synthase. *J Bacteriol*. 2007; **189:** 4299-4304.
24. Jogler C, Niebler M, Lin W, Kube M, Wanner G, Kolinko S et al. Cultivation-independent characterization of '*Candidatus* Magnetobacterium bavaricum' via ultrastructural, geochemical, ecological and metagenomic methods: *Candidatus* Magnetobacterium bavaricum. *Environ Microbiol*. 2010; **12:** 2466-2478.
25. Kaila VRI, Wikstrom M. Architecture of bacterial respiratory chains. *Nat Rev Microbiol*. 2021; **19:** 319-330.
26. Greening C, Biswas A, Carere CR, Jackson CJ, Taylor MC, Stott MB et al. Genomic and metagenomic surveys of hydrogenase distribution indicate H2 is a widely utilised energy source for microbial growth and survival. *ISME J*. 2016; **10:** 761-777.
27. Gross R, Simon J. The *hydE* gene is essential for the formation of *Wolinella succinogenes* NiFe-hydrogenase. *FEMS Microbiol Lett*. 2003; **227:** 197-202.
28. Koch H, Galushko A, Albertsen M, Schintlmeister A, Gruber-Dorninger C, Lucker S et al. Growth of nitrite-oxidizing bacteria by aerobic hydrogen oxidation. *Science*. 2014; **345:** 1052-1054.

29. Leung PM, Daebeler A, Chiri E, Hanchapola I, Gillett DL, Schittenhelm RB et al. A nitrite-oxidising bacterium constitutively consumes atmospheric hydrogen. *ISME J*. 2022; **16:** 2213-2219.

30. Friedrich CG, Rother D, Bardischewsky F, Quentmeier A, Fischer J. Oxidation of reduced inorganic sulfur compounds by bacteria: emergence of a common mechanism? *Appl Environ Microbiol*. 2001; **67:** 2873-2882.

31. Greene EA, Hubert C, Nemati M, Jenneman GE, Voordouw G. Nitrite reductase activity of sulphate-reducing bacteria prevents their inhibition by nitrate-reducing, sulphide-oxidizing bacteria. *Environ Microbiol*. 2003; **5:** 607-617.

32. Quast C, Pruesse E, Yilmaz P, Gerken J, Schweer T, Yarza P et al. The SILVA ribosomal RNA gene database project: improved data processing and web-based tools. *Nucleic Acids Res*. 2012; **41:** D590-D596.

33. Yu H, Leadbetter JR. Bacterial chemolithoautotrophy via manganese oxidation. *Nature*. 2020; **583:** 453-458.

34. Daims H, Nielsen JL, Nielsen PH, Schleifer KH, Wagner M. In situ characterization of *Nitrospira*-like nitrite-oxidizing bacteria active in wastewater treatment plants. *Appl Environ Microbiol*. 2001; **67:** 5273-5284.

35. Waterhouse AM, Procter JB, Martin DM, Clamp M, Barton GJ. Jalview Version 2--a multiple sequence alignment editor and analysis workbench. *Bioinformatics*. 2009; **25:** 1189-1191.
